# Lessons from the design, development and implementation of a three-dimensional (3D) neonatal resuscitation training smartphone application: Life-saving Instruction for Emergencies (LIFE app)

**DOI:** 10.1186/s41077-021-00197-7

**Published:** 2022-01-10

**Authors:** Conrad Wanyama, Shobhana Nagraj, Naomi Muinga, Timothy Tuti, Hilary Edgcombe, Anne Geniets, Niall Winters, Mike English, Jakob Rossner, Chris Paton

**Affiliations:** 1 KEMRI/Wellcome Trust Research Programme, Kenya Medical Research Institute, 197 Lenana Place, P.O Box 43640-00100, Nairobi, Kenya; 2 Nuffield Department of Women’s & Reproductive Health, Oxford, England; 3 The George Institute for Global Health, London, UK; 4 Oxford University Hospitals NHS Trust, Oxford, UK; 5 Department of Education, University of Oxford, Oxford, UK; 6 Nuffield Department of Medicine, University of Oxford, Oxford, UK

**Keywords:** Human-centred design, Medical education, Simulation-based education, Frontline health workers

## Abstract

Neonatal mortality remains disproportionately high in sub-Saharan Africa partly due to insufficient numbers of adequately trained and skilled front-line health workers. Opportunities for improving neonatal care may result from upskilling frontline health workers using innovative technological approaches. This practice paper describes the key steps involved in the design, development and implementation of an innovative smartphone-based training application using an agile, human-centred design approach. The Life-saving Instruction for Emergencies (LIFE) app is a three-dimension (3D) scenario-based mobile app for smartphones and is free to download. Two clinical modules are currently included with further scenarios planned. Whilst the focus of the practice paper is on the lessons learned during the design and development process, we also share key learning related to project management and sustainability plans, which we hope will help researchers working on similar projects.

**Table:** 

**Key points**	
1. Healthcare training app design should address important clinical problems and the needs of end-users. 2. Relevant educational theory should be used in app design and subsequent evaluation by making explicit the presumed pathways of learning. 3. Human-centred design shares similarities with participatory approaches used in healthcare research and can be used to build empathy with participants and enable democratic decision-making in the process of app development. 4. Early engagement of a wide range of stakeholders, including those from professional accreditation bodies for health professionals, is useful in the subsequent scale-up of apps. 5. Effective app design can promote user engagement, learning, knowledge retention and improve usability whilst addressing the personal and professional needs of end-users.	

## Introduction

The first 28 days of life are the most vulnerable period of a child’s life and account for approximately 47% of all under-five deaths [1]. The majority of neonatal deaths are due to intrapartum asphyxia, preterm birth, birth defects and infections [2]. The sustainable development goals (SDGs) have outlined a target of reducing neonatal mortality to at least 12 deaths per 1000 live births by 2030 [3]. This target will be hard to achieve in low-resource settings due to a lack of skilled birth attendants capable of performing neonatal resuscitation and poor-quality care at birth [1, 2]. In sub-Saharan Africa, neonatal mortality remains high (27 per 1000 live births) with wide regional disparities [4]. The provision of high-quality facility-based care for birth, and the first 24 hours after birth, is recommended as it is the most critical time for complications to present. Equipping front-line health workers (FLHWs) with the appropriate skills to provide timely essential newborn care, recognise a sick newborn and neonatal resuscitation is an important strategy to improve quality of neonatal care [5, 6].

## The context of neonatal care in Kenya

Neonatal care in Kenya is predominantly provided by general nurses with the support of non-specialist junior clinicians. These nurses are often understaffed, overworked and inadequately prepared to provide high-quality care [7–9]. In 2019, the neonatal mortality rate in Kenya was 20 deaths per 1000 live births [1]. Newborn resuscitation in Kenya is formally taught through a variety of face-to-face courses including Paediatric Advanced Life Support, Advanced Life Support in Obstetrics, Emergency Obstetric and Newborn Care; Emergency Triage, Assessment and Treatment Plus Admission Care (ETAT+) [10]; and the recently launched Newborn ETAT+ course, a Newborn Essential Solutions and Technologies (NEST360°) project [11]. The ETAT+ course, running face-to-face over five days for FLHWs (3 to 5 days for undergraduate medical students), has been shown to improve clinical knowledge, skills retention, teamwork, working memory and decision-making [12]. By 2020, across Kenya, over 10,000 health care workers have been trained in newborn resuscitation through ETAT+ [13]. Despite this success, opportunities for improving FLHW’s knowledge and competencies in essential neonatal care and resuscitation are hampered by the limitations of face-to-face training. Face-to-face training is costly for junior clinicians and nurses and inaccessible to FLHWs in rural areas and those facing significant service pressures [14]. Implementation at scale in countries such as Kenya, is further restricted by limited availability of adequately trained clinical faculty [14]. Another key concern is the lack of refresher training for those already trained—a factor that is associated with knowledge decay and loss of clinical and psychomotor skills [15]. We therefore argue that there is a need to develop alternative and/or complementary strategies to deliver innovative neonatal resuscitation and refresher training to help mitigate the significant challenges facing FLHWs in Kenya, and those working in similar contexts.

## A smartphone-based application to provide simulation-based education

Approximately 88% of Kenya’s population including the current generation of nurses, clinicians and healthcare students reported owning a smartphone in 2015 [16, 17], and using smartphone applications for educational and clinical interventions in health [18]. Due to the ubiquity of smartphone use by FLHWs in Kenya, technology-enhanced learning through the use of smartphone devices is a potential strategy for providing targeted training in neonatal resuscitation to FLHWs. Advantages of using smartphones for training are that they can be delivered anywhere, anytime and at low cost, enabling learning at the individual’s convenience [19–22].

Simulation-based education (SBE) is usually implemented in clinical skills labs using manikins, and/or at the bedside with real patients or actors. Depending on how it is implemented, SBE offers a varied degree of fidelity, clinical realism and transferability to real clinical practice [22–24]. However, innovative digital platforms now exist that can deliver SBE on desktop computers and smartphones [25]. To afford the benefits of realism and engagement, design and implementation of SBE through smartphones requires creativity. Considering the above requirements and using the principles of human-centred design (HCD), design-thinking (used in software development), and relevant educational theory, we created the LIFE app (a smartphone-based simulation training application). The clinical content in the LIFE app is based on the ETAT+ course which utilises a simulation-based education approach [26], an educational strategy common for life-support courses. ETAT+ is taught to FLHWs working in maternity, paediatric/neonatal settings in Kenya and other Sub-saharan Africa countries and is used as a strategy to disseminate national paediatric/neonatal clinical practice guidelines [10, 27].

We present our experience of using a theory-informed HCD approach for the design, implementation, and early scale-up of the LIFE app in Kenya, to aid other researchers involved in developing technology-enhanced learning approaches for health in similar contexts. Whilst our work started before the coronavirus disease 2019 (COVID-19) pandemic, we subsequently embarked on building COVID-19 training scenarios into the app in response to the emerging user needs.

## What is the LIFE application?

LIFE app is a smartphone application that delivers scenario-based training using interactive three-dimensional (3D) graphics to simulate a real hospital environment. Using the touchscreen user interface, learners can navigate through the 3D hospital, find relevant equipment, and simulate the steps to manage a neonatal emergency. The LIFE app is free to download, playable offline anywhere, anytime. It teaches FLHWs how to manage common neonatal emergencies, based on Kenyan clinical practice guidelines, through the use of ETAT+ scenarios (applicable to similar settings across Sub-saharan Africa). The LIFE app can be used as a stand-alone method of learning, in conjunction with face-to-face training (within a “flipped classroom” approach [28]), and to promote continuous professional development (CPD). LIFE app was accredited by the Nursing Council of Kenya in May 2020 as an individualised CPD activity for nurses through the Kenya Paediatric Nurses’ Chapter. CPD accreditation ensures that the LIFE app scenarios are clinically relevant for improving health worker competence and promoting patient safety [29]. The LIFE app offers an immersive, interactive and personalised learning experience through testing of learned material followed by provision of adaptive reflective feedback with further opportunities for repetitive learning [30, 31].

**Fig. 1:**
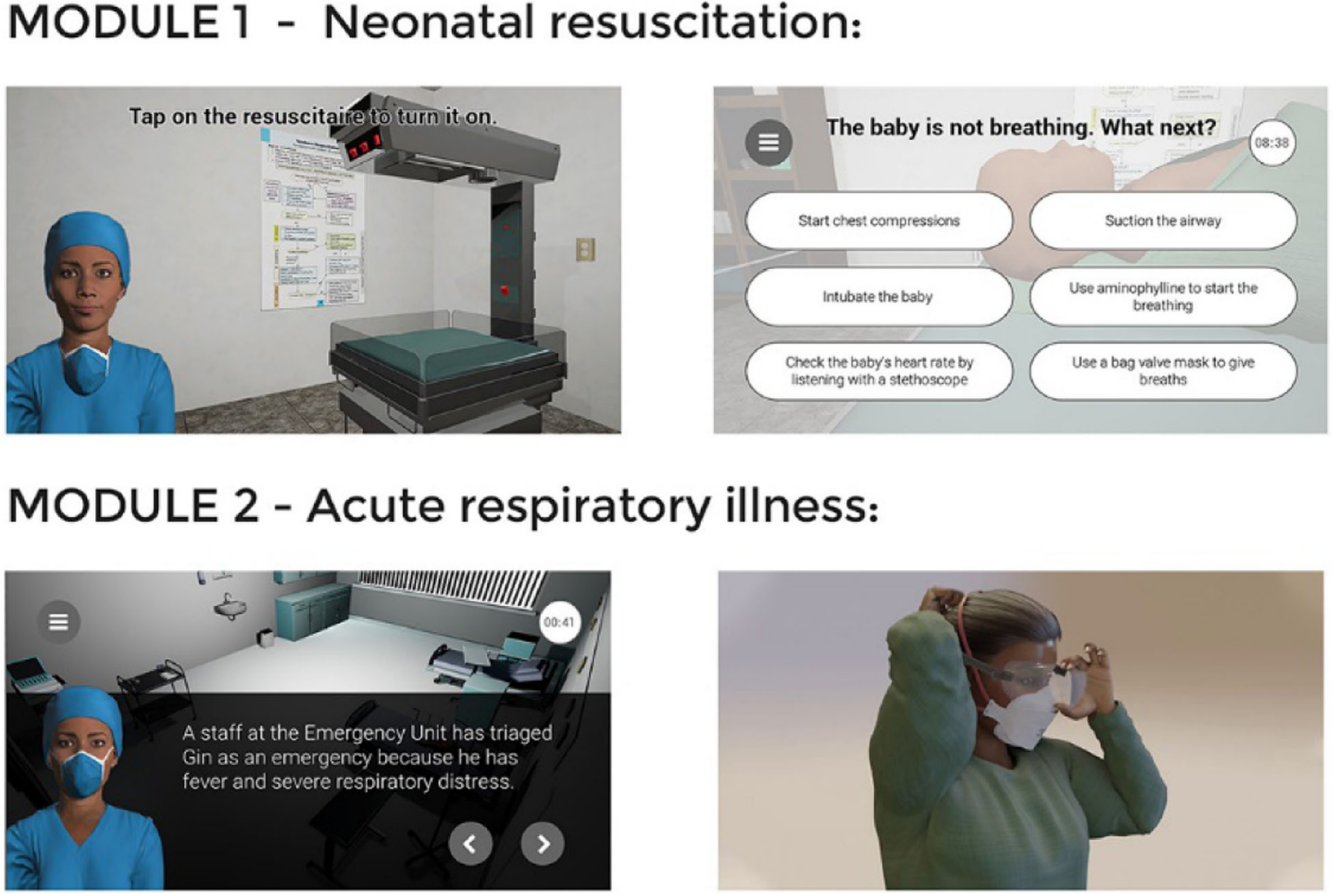
Screenshot images of LIFE 3D environment and scenarios for module one and two scenarios. This figure represents the LIFE-app images of one of the 3D interactive neonatal resuscitation scenario in use and the acute respiratory illness scenario under development (further information can be found at https://oxlifeproject.org/)

The LIFE app currently contains the neonatal resuscitation module with an additional acute respiratory illness (including COVID-19) under development. Each module is designed to host up to three simulated clinical scenarios of varying complexity. The scenarios in the neonatal resuscitation module teach FLHWs how to manage a range of neonatal emergencies in which the user takes on the role of a lone healthcare professional and has to resuscitate a newborn baby. The user must correctly answer a series of questions, find the right equipment in a virtual 3D ward environment, and then use the equipment to successfully resuscitate the baby. The acute respiratory illness module, once developed and availed to users, will aim at familiarising the FLHWs with the infection prevention and control measures for highly infectious respiratory illness such as COVID-19, as well as identification, classification and management of childhood pneumonia (Fig. 1).

## Development of the LIFE app

We now describe the design and development of the LIFE app in a step-wise approach, to enable future replication of our process.

### Identifying appropriate educational theories

Using a systematic search and review of instructional design features for technology-enhanced simulation-based education [32–34], we identified three relevant and synergistic educational theories: spaced repetition [35, 36], retrieval practice [30] and deliberate practice [37]), embedding them in the app design, thereby firmly grounding its contents within an educational epistemology.

The theory of spaced repetition describes how, following a learning encounter, human memory rapidly and continuously declines [31]. Providing opportunities for repetitive and spaced practice can reduce the rate of knowledge decline [37]. By frequent and adequately spaced review of previously learned material, memorisation of tasks is gradually improved [35–37]. The theory of retrieval practice recognises the importance of appropriate testing and feedback in influencing the recall of learnt material [32]. Finally, in order to train an individual to an expert level, systematic deliberate practice is required [37–39], otherwise learning rapidly plateaus and declines. The theory of deliberate practice focuses on targeting areas of weakness for learners, rather than further rehearsal of skills where competence has been achieved.

These three theories underpinned key design features of the LIFE app. Scenarios were designed to ensure repetitive plays and incremental learning. Testing and feedback were embedded into each clinical scenario, assessing learning and knowledge retention through multiple choice quizzes, and providing individualised feedback. Monthly CPD points were awarded via the app to encourage spaced (monthly) repetitions. A ‘remind, repeat and recall’ self-regulated learning plan was embedded in the app to encourage FLHWs to periodically refresh their training and test their knowledge. Thus, repetitive play, spaced repetition, [35, 36] use of quizzes and provision of individualised feedback [31, 40] to enable targeted deliberate practice [37], with rewards of CPD points were embedded into the app design to create a theory-driven learning experience.

### Using a human-centred design approach

**Fig. 2:**
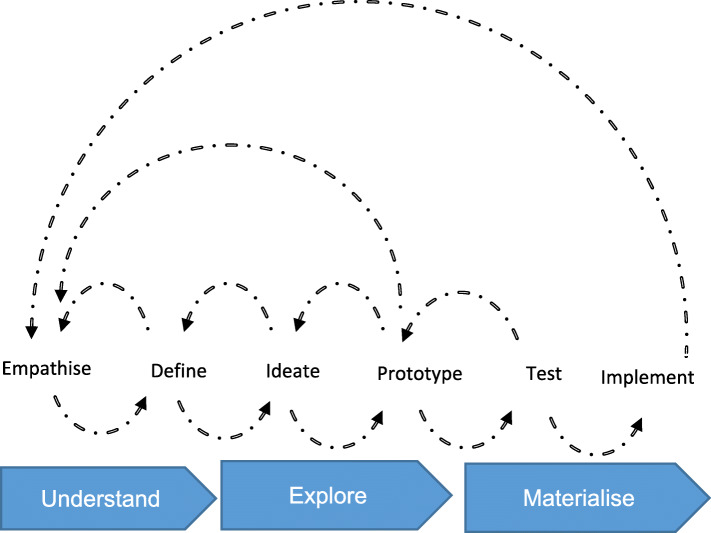
Schematic diagram to show the steps involved in iterative co-design, testing and implementing a prototype using a HCD approach. This is an illustration of the co-design iterative process that was used in the design of the LIFE app, emphasising the cyclic process for developing and refining the prototype with feedback from users as testers until the LIFE app is ready to be published on the Appstore/Playstore (adapted from Gibbons, 2016) [42]

Human-centred design (HCD) is ‘a process that ensures that the designs match the needs and the capabilities of the people for whom they are intended’ [41, 42]. HCD approaches marry well with participatory approaches used in health and social research. Both involve early and continuous stakeholder engagement, reflective and iterative learning and democratic decision-making [43, 44]. Fundamental principles of HCD include (i) understanding and addressing the root cause of problems to be addressed (not just the symptoms), (ii) developing a user-centred approach to meet users’ needs, (iii) adopting a systems approach where ‘all activities’ rather than ‘isolated components of the project’ are supported and (iv) using rapid iterative processes for designing and testing updates to the app (sprints). The iterative process emphasises refining the prototype through repeated feedback, implementing changes, and further user testing (Fig. 2) [45].

This cyclical process focuses on continuous learning, identification of problems and successes, and enlisting the strengths and experiences of the team to problem-solve until a product that meets the needs of users is fully developed.

We incorporated the principles of HCD into a flexible and dynamic process of LIFE app development. We engaged key stakeholders involved in the delivery or practice of neonatal resuscitation in Kenya (professional organisations, government officials, doctors, ETAT+ trainers, nurses, midwives and healthcare students) at each step of the process from ideation to design, and in user testing and app refinement. The HCD development of the LIFE app involved seven steps.

**Step 1:** User story-mapping through co-design workshops. Personas of FLHWs were developed and user stories were identified to inform the overall design of the LIFE app and what scenarios and features would meet their needs.

**Step 2:** Development of a Minimum Viable Product. The aim of this stage was to produce an app ready for initial iterative internal testing *(alpha testing)* by a small team of users (project team members composed of a developer, clinical content experts and project team leaders). The purpose of alpha testing is to identify and fix any software bugs, determine app stability and iteratively redefine the problem and change, improve or refine the app.

**Step 3:** Participatory testing and learning phase with nursing students from Oxford Brookes University, UK. We formed a monthly nominal group of end-users (Oxford Brookes nursing students and the LIFE app project team) and structured a step-wise *beta testing* with both new and previous users in each monthly cohort. Through an iterative process of design-build-test cycles, we developed a simple and aesthetically appealing design [43–46]. This initial user interface feedback was aimed at getting feedback from users in a high-income country before later feedback from users in a low-middle income country, although we did not plan to compare the nature of feedback from each of these settings.

**Step 4:** First round of beta testing in Nairobi, Kenya with end-users to continued testing, feedback and app refinement [46]. End-users included the LIFE app project team at Kenya Medical Research Institute-Wellcome Trust Research Programme (KEMRI-WTRP), and local clinicians (senior paediatricians, senior paediatric nurses, neonatal nurses and neonatologists) and academics (ETAT+ clinical instructors and university/college lecturers and tutors). We are yet to publish the qualitative experiences of users in Kenya (a low-middle income country) and how this compares with the user experiences from a high-income country.

**Step 5:** Second round of beta testing with FLHWs in Nairobi, Kenya, to evaluate user experiences on product stability and playability. We collected specific feedback on ease of download, size of the app, aesthetics (screen presentation, size of words, app widgets, cues and prompts), logical flow, relevance and correctness of clinical content. Users also provided feedback on simulation features, including utility of in-game and end-game feedback, difficulty of clinical content and the role of self-directed learning in current and future learning curves [31, 46].

**Step 6:** Qualitative data collection: We engaged clinical teachers and practitioners, as LIFE app users, from Kenya’s healthcare institutions to understand the challenges of effectively delivering large-scale neonatal resuscitation training in Kenya and how to integrate LIFE app within a training curriculum. We also wanted to understand how LIFE app can be used to offer continuous professional development (CPD) points from various health regulatory bodies in Kenya. The findings of this work have been used to describe how self-directed learning shapes future learning curves in Sub-Saharan Africa [31].

**Step 7:** Release of a public version product of the LIFE app, made available to all FLHWs in maternal, neonatal and paediatric health facilities. The LIFE app incorporated a wide range of feedback from end-users in Oxford and Kenya before being made available for free download as a public smartphone app on both android Playstore and iPhone operating system (iOS) Appstore. The official public version was launched in 2019 at the Kenya Paediatric Association conference, and further publicised at the Kenya Paediatric Nurses’ Chapter conference and Council of International Neonatal Nurses Conference/Second African Neonatal Nursing Conference.

### Implementation: Lessons for the roll-out and scalability of LIFE app

During the design and development process, clinical users requested that the LIFE app, as a training activity, should earn CPD points awarded to both nursing and medical practitioners in Kenya. The concept of CPD in promoting life-long learning has two main purposes: (i) to bridge the knowledge, competence and performance gap for practitioners and (ii) to continually prepare practitioners to provide up-to-date, safe care for patients [47]. We therefore engaged two agencies: The Kenya Paediatric Association, (a certified CPD provider and professional association for physicians) and The Nursing Council of Kenya (the national regulator for nurses). The aim of the engagement was to review the content on the LIFE app, quantify the number of CPD points that could be awarded for each activity and define how individual practitioners would earn/claim CPD points. By aligning the LIFE app with recognised CPD providers in response to user feedback, we could scale the roll-out of the LIFE app, thereby creating demand from practitioners.

We also explored the opportunity for LIFE app to provide up-to-date clinical content based on national practice guidelines through regular app updates and knowledge and procedural level [48]. The LIFE app, therefore, further became a tool for the dissemination of updated local clinical practice guidelines. It was also proposed by some users (mainly ETAT+ teaching faculty) to embed LIFE app in the delivery of ETAT+ training. We saw the opportunity of using the LIFE app within a blended learning approach with ETAT+ training, whereby LIFE app content is offered ahead of the face-to-face training. This has potential to improve the educational effectiveness of the face-to-face training, reduce the time spent in delivering face-to-face training and its associated costs, whilst ensuring content was provided in a convenient way for end-users.

### The importance of effective project management

One of the keys to the successful development and implementation of the LIFE app has been effective project management. This is often overlooked in the research literature, and we present here how we employed project management skills to ensure team cohesion across two geographically distant sites.

**Fig. 3:**
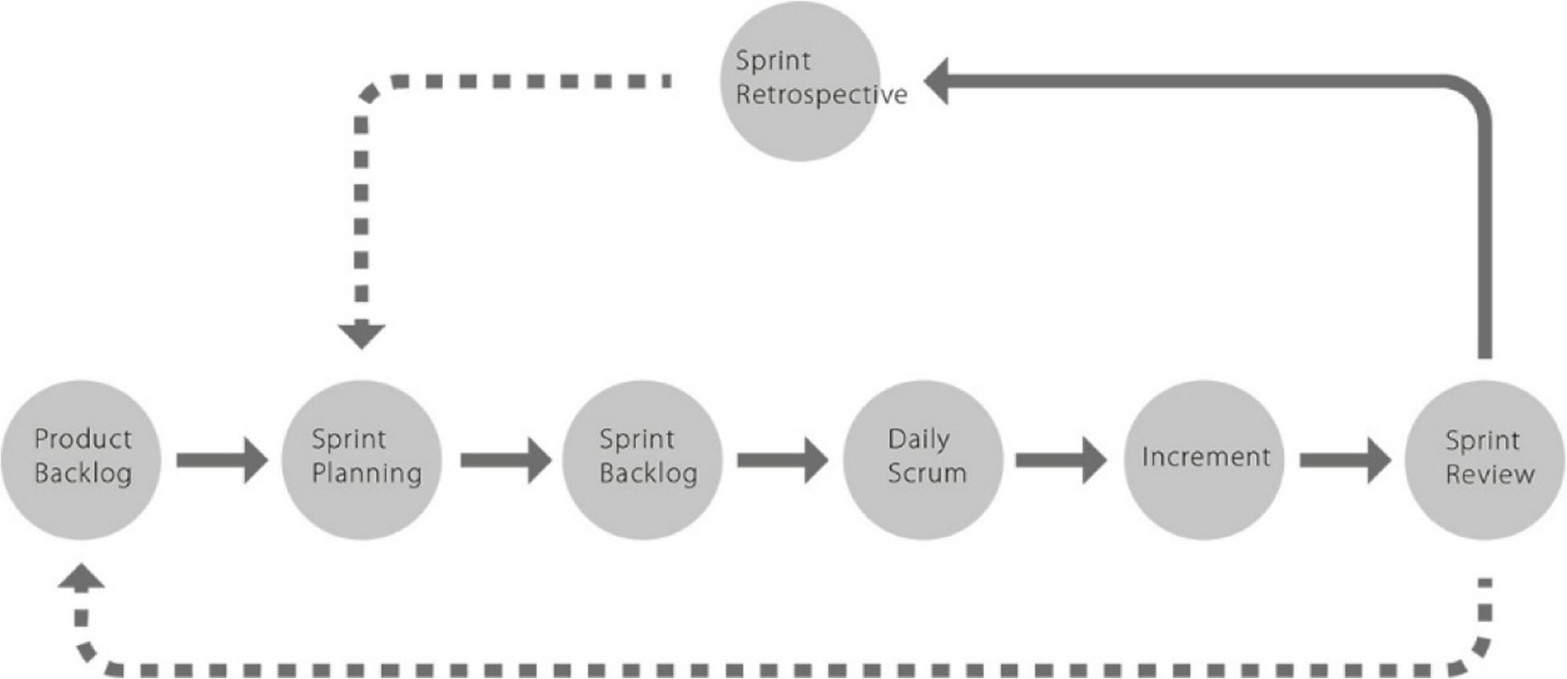
Schematic presentation of using the Scrum agile management framework. This is an image of the Asana© project management tool that employs Scrum methodology and how the tool was used during the LIFE app project duration

The aim of project management is to efficiently and effectively manage the available resources and produce the desired output. We employed the Agile software development values and principles that focus on collaborative team efforts and iterative ‘Scrum’ methods to produce a working software product [49]. Scrum is an iterative, agile management framework methodology that promotes incremental development of an app (Fig. 3).

Using Scrum methodology, we documented our project plans and activity milestones on a shared web-based project management tool (Asana©: www.asana.com) to plan and execute weekly team meetings for task allocation and daily ‘stand-up’ meetings for evaluating progress of the LIFE app project [49].

Meetings were attended face-to-face and virtually, by the LIFE app project co-design team (composed of clinicians, researchers and the lead developer) throughout the design phase to brainstorm potential problems and propose solutions. Using Asana©, we classified tasks into several categories: completed tasks, sprint tasks (need to be completed urgently), tasks in progress, ideas to be implemented (backlog) and those that require high-level decision-making (strategy tasks). Through weekly virtual meetings, we prioritised and allocated tasks to team members, whilst embedding the principles of HCD and iterative Scrum software development.

Although the LIFE app project team used the ‘Scrum’ methodology, employing the Asana© software tool, our recommendation is that similar app development projects should (i) set up a project team with clearly defined roles for each team member, (ii) clearly communicate the project goal(s) to the team members, with distinct but inter-related project activities (iii) engage various stakeholders, including users early in the co-design, development and implementation cycles of the product and (iv) utilise one agreed upon software tool that employs an iterative project design that enables the team to have internal team meetings for regular communication, task allocation, review of performance and one that can easily be integrated into work/institutional communication platforms.

### Financial models to develop and sustain use of the LIFE app

The initial project was funded through crowdfunding through Oxford University Innovation, University of Oxford. Sustainability plans for funding included grant applications for developing more clinical modules (such as the COVID-19 module) and subsequently availing these new modules as a package on the LIFE app used for CPD activities. Future initiatives include developing partnerships with relevant institutions to develop further training scenarios.

The clinical content (plus the 3D interactive features, such as the clinical environments, user navigations and clinical animations) has been designed to allow for future edits for purposes of making rapid updates at minimal costs.

Although apps are commonly paid up in high-income settings, this is not the case in low-middle-income settings, so the sustainability models might therefore differ. We, therefore, intend to avail, at no cost, the LIFE app to users in low- and middle-income countries, with the cost of running the updates on the LIFE app met by the professional health agencies as a sustainability initiative. The specific professional health agencies should be involved in developing, reviewing and updating local clinical practice guidelines as well as implementing the already existing ETAT+ training. We anticipate that these agencies (in our setting, the Kenya Paediatric Association and the University of Nairobi) will be the ones providing funding for regular content update on the LIFE app and availing it as an aspect of the blended ETAT+ course (pre-course smartphone LIFE app, followed by the face-to-face course and thereafter the post-course smartphone LIFE app).

## Conclusion

To date, there have been over 6000 app downloads. We plan to now focus on collecting qualitative data on the perceived user experiences and later on assess the effectiveness of LIFE app is now accredited by the Nursing Council of Kenya as a CPD activity for Kenyan nurses/midwives. We believe the successful development and dissemination of the app was based on the theory-informed and HCD approach to design and development, with early and continuous engagement of key stakeholders involved in the training and education of FLHWs in Kenya. User feedback was key to designing a practical strategy for roll-out and scalability. Lastly, we have described the importance of employing project management principles to manage available resources, with the goal of producing a high-quality, user-centred product. Our future work will further look at adaptive learning tailored to provide a personalised learning experience for users [40].

## Data Availability

Not applicable.

## Abbreviations

COVID-19: Coronavirus disease 2019
CPD: Continuous professional development
ETAT+: Emergency triage assessment and treatment plus admission care
FLHWs: Front-line health workers
HCD: Human-centered design
KEMRI: Kenya medical research programme
LIFE app: Life-saving instruction for emergencies application
SBE: Simulation-based education
